# TIMM8A-TIMM13 Complex Exerts Oncogenic Functions in Lung Cancer

**DOI:** 10.32604/or.2025.063812

**Published:** 2025-08-28

**Authors:** Shengmin Li, Kejian Shi, Ying Wang, Yi Zhang

**Affiliations:** 1 Department of Thoracic Surgery, Xuanwu Hospital, Capital Medical University, Beijing, 100053, China; 2 National Center for Orthopaedics, Department of Molecular Orthopedics, Beijing Research Institute of Traumatology and Orthopaedics, Beijing Jishuitan Hospital, Capital Medical University, Beijing, 100035, China

**Keywords:** Lung cancer, prognosis, proliferation, TIMM8A-TIMM13 complex, CCND1-CDK6 complex

## Abstract

**Objectives:**

Lung cancer represents a major global healthcare challenge, characterized by high annual incidence and mortality rates worldwide. Although targeted therapies for lung cancer have advanced, treatment outcomes for advanced-stage patients remain suboptimal. This investigation examines the role of the translocase of the inner mitochondrial membrane (TIMM)8A-TIMM13 complex in lung cancer and evaluates its potential as a novel therapeutic target.

**Methods:**

A co-immunoprecipitation (Co-IP) assay was conducted to verify the interaction between TIMM8A and TIMM13. Differential gene expression analysis of TIMM8A or TIMM13 was executed using the TNMplot database, with survival estimates derived from the Kaplan-Meier plotter. Lung cancer cell proliferation was evaluated through Cell Counting Kit 8 (CCK-8) and colony formation assays, while cell migration was assessed via Transwell assay. RNA sequencing identified the downstream effectors of TIMM13. RNAi technology facilitated the inhibition of TIMM8A or TIMM13 expression, which was measured through immunoblotting or qRT-PCR.

**Results:**

This investigation revealed that components of the TIMM8A-TIMM13 complex exhibited elevated expression in human lung cancer tissues, correlating with disease progression and poor overall survival rates among lung cancer patients. The suppression of either TIMM8A or TIMM13 inhibited cell proliferation and migration. Mechanistic studies through transcriptome analysis identified cell cycle-related pathways as potential key downstream effectors of the TIMM8A-TIMM13 complex. Subsequent experiments confirmed that the TIMM8A-TIMM13 complex significantly regulated the expression of cyclin D1 (CCND1) and cyclin-dependent kinase 6 (CDK6) complex.

**Conclusion:**

The elevated expression of TIMM8A-TIMM13 complex components plays a crucial role in lung cancer cell growth, suggesting its potential as a promising therapeutic target for lung cancer treatment.

## 1 Introduction

Lung cancer represents the second most frequently diagnosed cancer globally, with 2.2 million new cases reported in 2020 [1], posing a significant threat to public health. Despite considerable advances in therapeutic options, lung cancer remains the leading cause of cancer-related mortality worldwide [1]. Non-small cell lung cancer (NSCLC) constitutes the predominant form, representing 80%~85% of all lung cancer cases [2]. Notably, approximately two-thirds of NSCLC patients present with advanced-stage disease at diagnosis [3]. The prognosis for advanced-stage NSCLC remains unfavorable, with a mere 15% five-year survival rate for these patients [4]. Given that the molecular mechanisms underlying the proliferative and invasive characteristics of lung cancer remain incompletely understood, further investigation of these mechanisms is essential for improving treatment strategies and patient outcomes.

Beyond their fundamental role as primary ATP generators via oxidative phosphorylation, mitochondria function as multifunctional signaling hubs coordinating diverse physiological processes, including calcium homeostasis, cell cycle regulation, redox balance, and apoptosis [5,6]. Mitochondrial quality control is maintained through coordinated mechanisms including mitochondrial biogenesis, mitophagy, dynamic membrane remodeling via fusion and fission, and transcriptional control of mitochondrial genes [7]. Emerging evidence indicates that dysregulated mitochondrial homeostasis contributes to multiple disorders, including lung cancer. Notably, elevated mitochondrial functions have been demonstrated to drive tumorigenesis, metastasis, and therapy resistance in malignancy [8]. For instance, the mitochondrial matrix-localized OTUB1 (OTU domain-containing ubiquitin aldehyde-binding protein 1), a negative regulator of degradative ubiquitination that governs the stability of intramitochondrial proteins, is frequently over-expressed and plays a pro-oncogenic role in lung tumors [9], making it a promising candidate for lung cancer therapy. Other studies have recently shown that HSP60 (heat shock protein 60), a chaperone mainly localized in the mitochondrial matrix where it accounts for the transportation and refolding of proteins from the cytoplasm directly into the mitochondrial matrix, has been found to be commonly over-expressed in NSCLC and associated with poor prognosis [10,11].

The maintenance of mitochondrial functions largely depends upon the biogenesis of the inner mitochondrial membrane, which relies on the coordinated activities of distinct protein import machinery [12]. The translocase of the inner mitochondrial membrane (TIMM) 8A-TIMM13 complex in the mitochondrial intermembrane space represents an essential component of the import machinery involved in the import and assembly of the TIMM23 complex in the inner mitochondrial membrane, proper biogenesis of which is indispensable for the import of all matrix proteins as well as many inner mitochondrial membrane proteins [13]. Given mitochondria’s critical role in cellular survival, aberrant mitochondrial import and assembly complexes potentially impact human health significantly. Recent research demonstrates that TIMM8A functions as an oncogene in breast cancer and correlates positively with poor prognosis in breast cancer patients [14–17]. Similarly, TIMM13 has been recently identified to be associated with tumorigenesis in some certain malignancies, including skin cutaneous melanoma and osteosarcoma [18,19]. However, the roles and mechanisms of TIMM8A-TIMM13 complex in lung cancer remain poorly understood.

Based on the emerging role of mitochondrial protein import dysregulation in cancer progression and the reported oncogenic functions of the TIMM8A-TIMM13 complex in other malignancies, we hypothesized that this complex might be involved in the development of lung cancer. This study aimed to (1) validate the clinical relevance of TIMM8A-TIMM13 complex in lung cancer, (2) elucidate its functional impact on lung cancer cell proliferation and migration, and (3) identify its downstream effectors, to assess its therapeutic potential.

## 2 Materials and Methods

### 2.1 Reagents and Antibodies

The following reagents were utilized in this study: Dulbecco’s modified Eagle medium (DMEM) (Wisent, 319-005-CL, Saint-Jean-Baptiste, QC, Canada); fetal bovine serum (FBS) (Wisent, 087-150, Saint-Jean-Baptiste, QC, Canada); phosphate buffer saline (PBS) (Gibco, C10010500BT, Thermo Fisher, Waltham, MA, USA); penicillin and streptomycin (PS) (Gibco, 450-201-EL); Cell Counting Kit 8 (CCK-8) (Dojindo, CA1210, Kumamoto, Japan); TRIzol reagent (Invitrogen, 15596018CN, Thermo Fisher, Waltham, MA, USA); Lipofectamine^TM^ 2000 transfection reagent (Invitrogen, 11668027); the siRNA oligonucleotides synthesized by GenePharma company (Suzhou, China); primers for qPCR synthesized by Sangon Biotech (Shanghai, China); PrimeScript RT Master Mix (TaKaRa, RR036A, Beijing, China); Pierce^TM^ RIPA buffer (Thermo Fisher Scientific, 89900, USA); TIMM8A polyclonal antibody (Proteintech Group, 11179-1-AP, Wuhan, China); TIMM13 polyclonal antibody (Proteintech Group, 11973-1-AP); cyclin D1(CCND1) polyclonal antibody (Proteintech Group, 26939-1-AP); CDK6 polyclonal antibody (Proteintech Group, 19117-1-AP); β-actin mouse mAb (Beyotime Biotechnology, AF0003, Shanghai, China); horseradish peroxidase (HRP)-conjugated goat anti-mouse IgG (ZSGB Biotech, ZB-5305, Beijing, China); HRP-conjugated goat anti-rabbit IgG (ZSGB Biotech, ZB-2301); HRP-conjugated polymer secondary antibody (ZSGB, Biotech, PV6000); 3,3^′^-diaminobenzidine (DAB) (ZSGB Biotech, ZLI9017); Mayers’ Hematoxylin Solution (Sigma-Aldrich, MHS16, St. Louis, MO, USA); Protein A/G Agarose (Gene-Protein Link, GPL2003, Beijing, China); and cOmplete ULTRA Tablets, Mini, EDTA-free, EASYpack (Roche, 05892791001, Basel, Switzerland).

### 2.2 Clinical Specimens

Lung tumor tissues and paired adjacent non-tumor tissues were collected from lung cancer patients who underwent surgery at Xuanwu Hospital between January 2023 and October 2023. None of the patients underwent chemotherapy or radiotherapy before surgical intervention. All patients provided informed consent, and the research was approved by the ethical committee of Xuanwu Hospital (Approval No. 2022162). The tissues were immediately frozen in liquid nitrogen post-resection and underwent pathological diagnosis.

### 2.3 Bioinformatic Analyses

The TNMplot database was utilized to perform differential gene expression analysis of the TIMM8A-TIMM13 complex in normal, tumor, and metastatic tissues [20]. The Gene Expression Profiling Interactive Analysis (GEPIA) database was employed to examine the correlation between TIMM8A or TIMM13 mRNA levels and tumor stages in lung cancer [21]. Survival analyses were conducted using Kaplan-Meier plotter [22] for lung cancer.

### 2.4 Cell Culturing

Two lung cancer cell lines, A549 and PC-9, were obtained from the Chinese Academy of Sciences Shanghai Cell Bank. Short tandem repeat (STR) profiling was used to authenticate all cell lines, and mycoplasma contamination testing was conducted to confirm their sterility. Both cell lines were grown in a complete DMEM medium (10% FBS and 1% penicillin/streptomycin) at 37°C in a 5% CO_2_ environment.

### 2.5 RNA Interference

After plating cells in 6-well plates, gene silencing was achieved through Lipofectamine^TM^ 2000-mediated siRNA transfection following the manufacturer’s instructions. siRNA targeted sequences are as follows: TIMM8A-66: 5^′^-CATCGAGGTAGAGACTCAA-3^′^; TIMM8A-228: 5^′^-CATCTTGAATCGACTGGAA-3^′^; TIMM13-118: 5^′^-CAGAGGATGACGGACAAGT-3^′^; TIMM13-215: 5^′^-GCTACATGGACGCCTGGAA-3^′^; negative control (NC): 5^′^-TTCTCCGAACGTGTCACGT-3^′^.

### 2.6 Cell Counting Kit-8 (CCK-8)

Cell proliferation was assessed using CCK-8 assay according to the manufacturer’s protocol. Cells were seeded in quadruplicate into 96-well plates at 5 × 10^3^ cells per well and cultured overnight. Following transfection with the indicated siRNA, cells were cultured for up to 96 h. At specified time points, cells were incubated with CCK-8 agent in DMEM for 1 h at 37°C. Measurements were performed at 450 nm for detection and 600 nm for background subtraction.

### 2.7 Colony-Formation Assay

Cells were seeded in 6-well plates at a density of 500 cells per well. After two weeks, the cells were fixed using 90% ethanol for 10 min and then stained with 0.1% crystal violet for 5 min. Following three washes with PBS and air-drying, the colonies were examined and counted manually.

### 2.8 Transwell Assay

Approximately 5 × 10^4^ cells were cultured in FBS-free DMEM medium in transwell chambers, which were positioned in a 24-well plate containing DMEM medium supplemented with 10% FBS. After approximately 10 h, cells in the transwell chambers were fixed with 90% ethanol for 10 min and stained with 0.1% crystal violet for 5 min. Cells on the lower side of the transwell chambers were subsequently examined.

### 2.9 Immunoblotting

Immunoblotting analysis was conducted as previously described [23]. Briefly, total cell lysates (approximately 30 μg per lane) underwent immunoblotting using primary antibodies against TIMM8A (1:1000), TIMM13 (1:1000), Cyclin D1 (1:2000) and anti-CDK6 (1:2000) at 4°C overnight, followed by incubation with secondary antibodies (1:5000) for two hours at room temperature. β-actin (ACTB) served as the internal control.

### 2.10 Immunohistochemistry (IHC)

The patient’s tumor and adjacent non-tumor samples underwent deparaffinization in xylene and rehydration through graded ethanol. Slides were subjected to heat-induced antigen retrieval using citrate buffer (pH 6.0) at 95°C–100°C for 20 min. After blocking the activity of endogenous peroxidase with 3% H_2_O_2_ and nonspecific binding with 5% normal serum, sections were incubated with the primary antibody (the dilution ratio of TIMM8A Polyclonal antibody: 1:400; the dilution ratio of TIMM13 Polyclonal antibody: 1:200) overnight at 4°C, followed by incubation with an HRP-conjugated polymer secondary antibody (undiluted) for 30 min at room temperature. Signals were developed using 3,3^′^-diaminobenzidine (DAB) substrate, counterstained with hematoxylin (Sigma-Aldrich, MHS16, USA), dehydrated through graded ethanol, cleared in xylene, and mounted for microscopic analysis. ImageJ 1.54 g (Wayne Rasband and contributors National Institutes of Health, USA) was utilized to measure integrated optical density for quantitative analysis.

### 2.11 RNA Sequencing

Total RNA from A549 lung cancer cells was extracted using TRIzol according to the manufacturer’s instructions. The RNA quality was rigorously assessed using an Agilent Fragment 5300 Analyzer system (Agilent Technologies, Santa Clara, CA, USA), with all experimental samples achieving RNA Integrity Number (RIN) scores of 10. Subsequently, the qualified RNA samples were used to construct a qualified single-stranded circular DNA library, which underwent sequencing using the DNBSEQ^TM^ platform by the Beijing Genomics Institution. All data analysis was performed using the BGI Dr. Tom system (https://biosys.bgi.com/, accessed on 5 June 2025).

### 2.12 Co-Immunoprecipitation (Co-IP)

A total of 8 × 10^6^ A549 cells were collected and washed. The cells underwent incubation with Pierce^TM^ RIPA buffer containing protease inhibitor for 30 min. Each sample was incubated with antibodies at 4°C overnight. Following incubation with Protein A/G Agarose at 4°C for approximately 4 h, the sample underwent three PBS washes. Finally, an SDS loading buffer was added and the mixture was heated at 95°C for approximately 10 min.

### 2.13 Quantitative Reverse Transcription PCR (qRT-PCR)

Total RNA was harvested for qRT-PCR analysis as described previously [23]. Oligonucleotide primers were synthesized to detect TIMM8A and TIMM13 with GAPDH as internal control. The human-specific primer sequences are as follows: TIMM8A-forward: 5^′^-TCGTCTCTGCAAGCTTGGTC-3^′^, TIMM8A-reverse: 5^′^-TCCATGCACTTCTCCCAACAA-3^′^; TIMM13-forward: 5^′^-TCACCCTGTTGGGGAGAGAA-3^′^, TIMM13-reverse: 5^′^-CAGCCGTTTATTGGGCAACC-3^′^; GAPDH-forward: 5^′^-AAGGTCGGAGTCAACGG-3^′^, GAPDH-reverse: 5^′^-GGAAGATGGTGATGGGATT-3^′^.

### 2.14 Statistical Analysis

All data were presented as mean ± standard deviation (*n* = 3 independent replicates for *in vitro* experiments; *n* = 18 for IHC analysis). One-way analysis of variance (ANOVA) was conducted to compare means among groups. Subsequently, multiple comparisons were performed using Fisher’s least significant difference (LSD) multiple comparisons test. Prior to ANOVA, the Shapiro-Wilk test assessed data normality while Bartlett’s test evaluated variance homogeneity. Comparisons between the two groups were conducted using the independent-sample *t*-test. The integrated optical density (IOD) of the two groups was analyzed using a paired *t*-test. A *p*-value < 0.05 was deemed statistically significant. Statistical analyses were carried out with GraphPad Prism version 8.0 (GraphPad, Boston, MA, USA).

## 3 Results

### 3.1 Bioinformatics Analysis of TIMM8A and TIMM13 Expression in Lung Cancer and Their Correlation with Patient Survival

**Figure 1:**
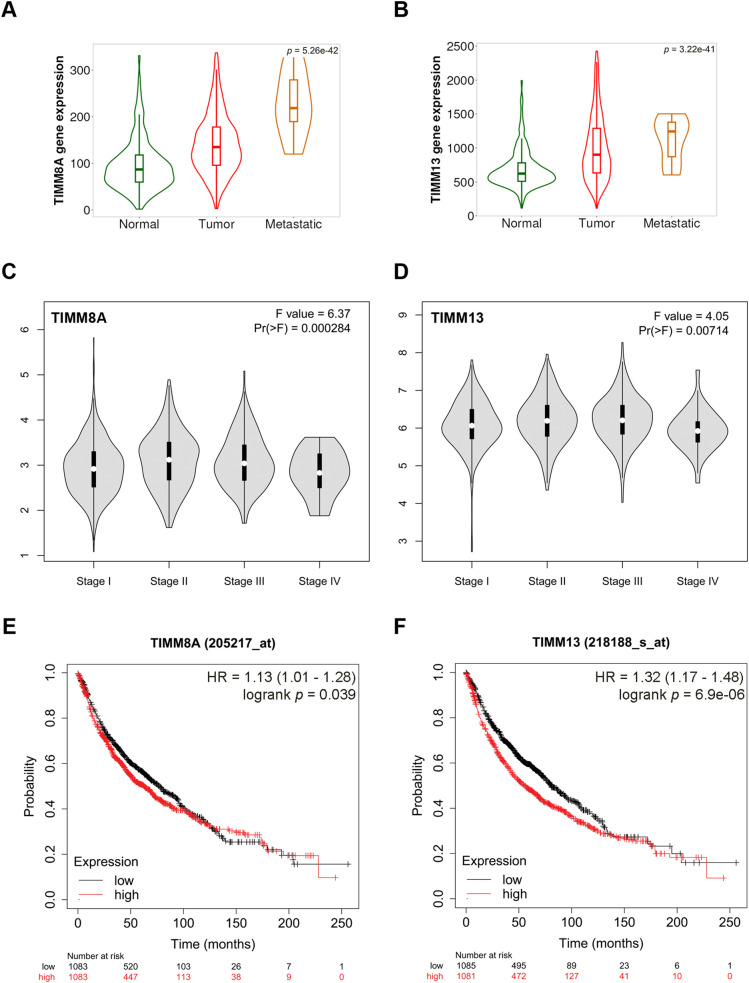
Overexpression of components of the mitochondrial TIMM8A-TIMM13 complex and their association with disease progression and clinical outcomes in lung cancer. (**A**, **B**) The expression of TIMM8A and TIMM13 is based on the TNMplot database. The mRNA expression level of either TIMM8A or TIMM13 in tumor tissues was higher than that in normal lung tissues (TIMM8A, Dunn test. *p* = 8.56e−42; TIMM13 Dunn test. *p* = 2.31e−42), while lower than that in metastatic tissues (TIMM8A, Dunn test. *p* = 6.85e−03; TIMM13 Dunn test. *p* = 1.66e−01). (**C**, **D**) The relationship between the mRNA level of either TIMM8A or TIMM13 and lung cancer disease stages was established with GEPIA. (**E**, **F**) Kaplan-Meier overall survival curves of human lung cancer patients with low vs. high TIMM8A/TIMM13 expression. The generated *p*-value does not include correction for multiple hypothesis testing by default

To investigate the potential role of TIMM8A-TIMM13 complex in lung cancer, the clinical relevance of TIMM8A and TIMM13 expression levels in lung cancer was initially evaluated. As shown in Fig. 1A,B, violin plots based on the TNMplot database demonstrated that mRNA levels of both TIMM8A and TIMM13 correlate positively with advanced disease (TIMM8A, Kruskal-Wallis. *p* = 5.26e−42; TIMM13 Kruskal-Wallis. *p* = 3.22e−41). Similarly, according to the GEPIA database, mRNA levels of both TIMM8A and TIMM13 showed a positive association with the disease stage (Fig. 1C,D). Subsequently, Kaplan-Meier overall survival rates in lung cancer patients were analyzed using a web-based survival analysis tool. The analysis revealed that lung cancer patients with high expression of either TIMM8A or TIMM13 exhibited a worse overall survival rate (Fig. 1E,F). These findings indicate that both TIMM8A and TIMM13 are over-expressed and associated with disease progression and poor clinical outcomes in lung cancer.

### 3.2 IHC Validation of the Expression Pattern of TIMM8A-TIMM13 Complex in Lung Cancer

**Figure 2:**
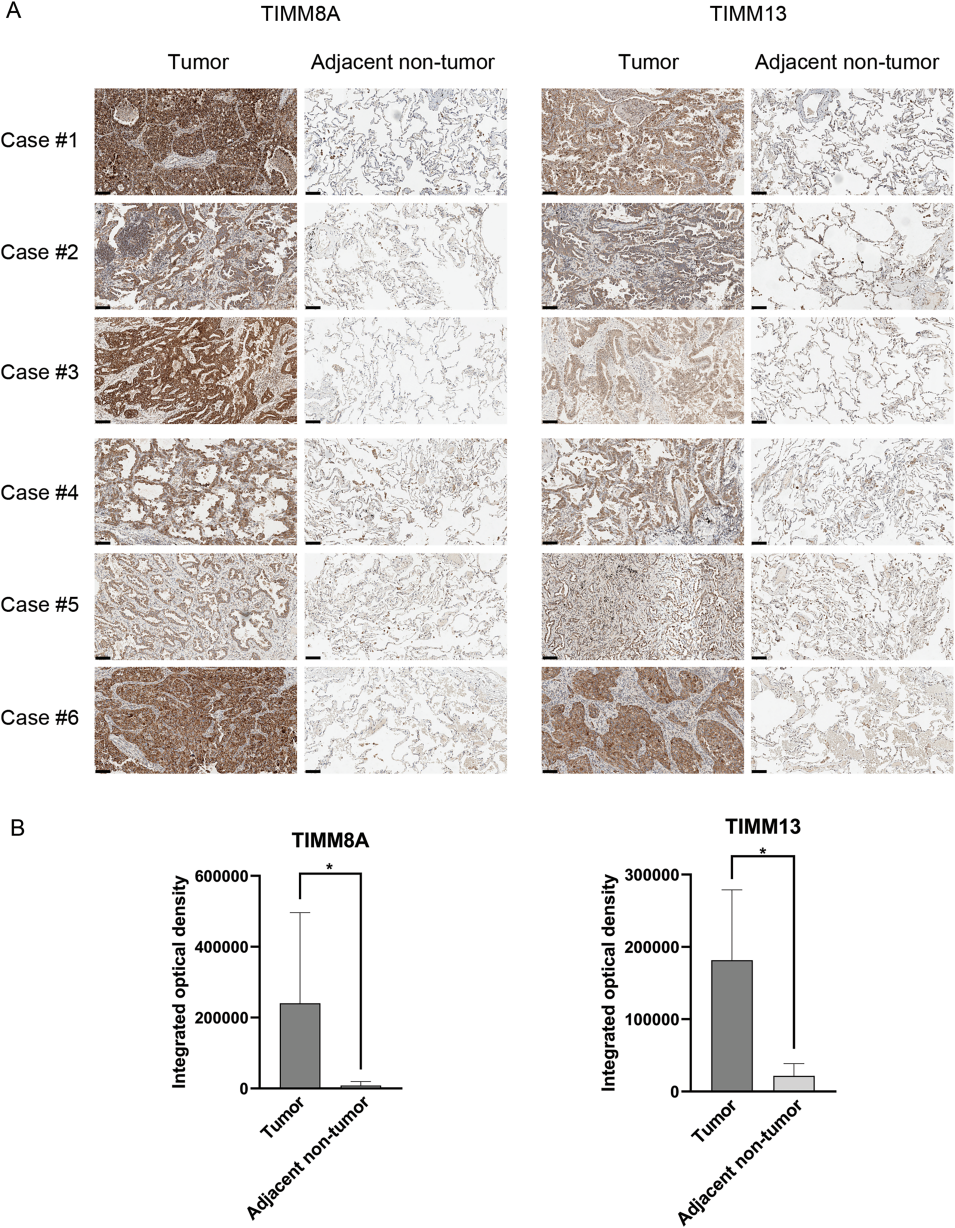
Verification of TIMM8A and TIMM13 expression in clinical samples. Representative IHC images (**A**) and IHC scores (**B**) of TIMM8A and TIMM13 expression in 18 lung cancer tissues and paired adjacent normal tissues (Scale bar = 50 μm), *p < 0.05

To confirm the expression pattern of TIMM8A-TIMM13 complex in lung cancer, the expression levels of TIMM8A and TIMM13 were evaluated in clinical tumor samples and paired adjacent non-tumor lung tissues using IHC analysis. As illustrated in Fig. 2A,B, and Supplementary Fig. S1, the expression levels of both TIMM8A and TIMM13 showed elevation in tumor tissues compared to the paired normal tissues. These observations demonstrate that both TIMM8A and TIMM13 were dysregulated in lung cancer.

### 3.3 Suppression of Either TIMM8A or TIMM13 Inhibited the Growth and Migration of Lung Cancer Cells

**Figure 3:**
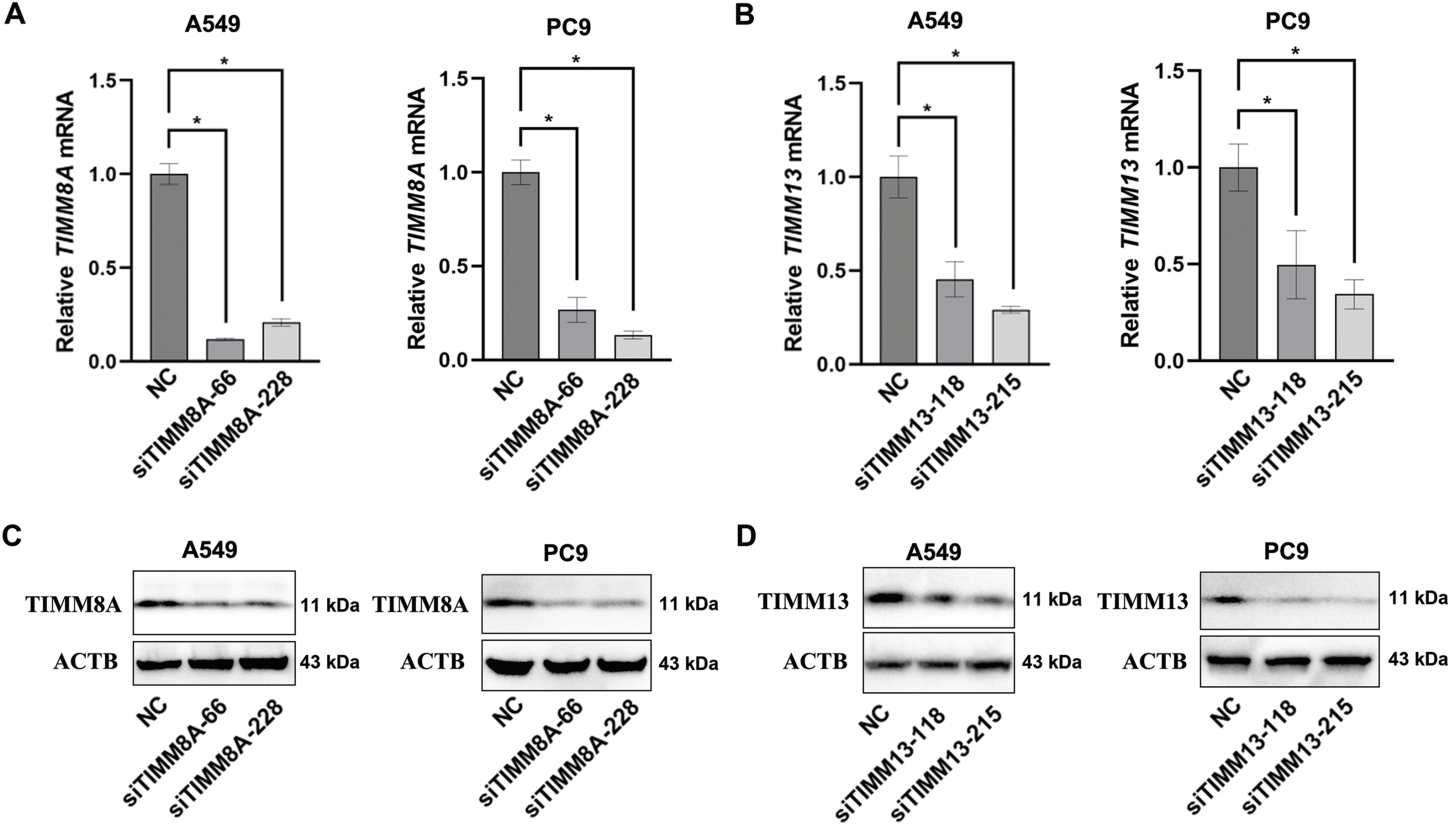
Transfecting siRNA could down-regulate the expression of TIMM8A or TIMM13. (**A**, **B**) The downregulation of TIMM8A or TIMM13 in A549 and PC9 cells was quantified using qRT-PCR. Results are presented as mean ± standard deviation, with *p* < 0.05 indicating statistical significance (*n* = 3). (**C**, **D**) The reduction of TIMM8A or TIMM13 expression in A549 and PC9 cells was verified by immunoblotting. *p < 0.05.

**Figure 4:**
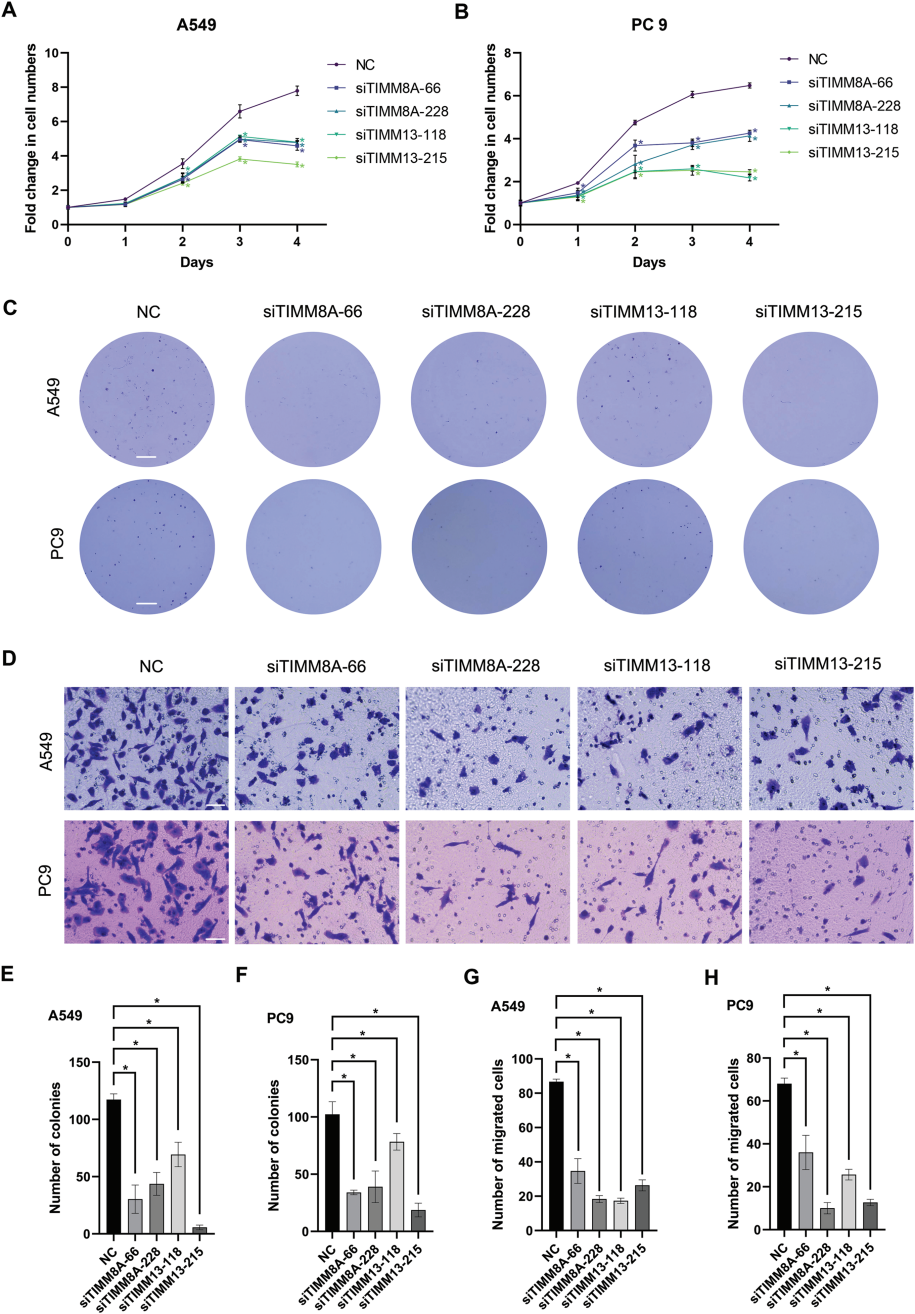
Inhibition of either TIMM8A or TIMM13 suppressed the growth and migration of lung cancer cells. (**A**, **B**) CCK-8 assay demonstrated significantly reduced cell viability following TIMM8A or TIMM13 knockdown in A549 (**A**) or PC9 (**B**) cells. (**C**) TIMM8A or TIMM13 knockdown diminished colony-forming capacity in A549 and PC9 cells. Scale bar = 4 mm. (**D**) Downregulation of either TIMM8A or TIMM13 reduced the migration capacity of A549 and PC9 cells. Scale bar = 50 μm. (**E**, **F**) Quantification of crystal violate-stained colonies formed by A549 (**E**) or PC9 (**F**) cells. (**G**, **H**) Quantification of crystal violate-stained migrated A549 (**G**) or PC9 (**H**) cells. Data are presented as mean ± standard deviation (*n* = 3). *p < 0.05 vs. NC

To evaluate the effects of upregulated expression of TIMM8A or TIMM13 on the biological behaviors of lung cancer cells, two distinct siRNAs targeting either TIMM8A (siTIMM8A-66 and siTIMM8A-228) or TIMM13 (siTIMM13-118 and siTIMM13-215) were employed to silence TIMM8A or TIMM13 in two lung cancer cell lines, A549 and PC-9. Initially, qRT-PCR analysis was performed to confirm the downregulated mRNA level of TIMM8A and TIMM13 in A549 and PC-9 cells (Fig. 3A,B). Subsequently, immunoblotting analysis verified the decreased TIMM8A or TIMM13 expression in both A549 and PC-9 cells (Fig. 3C,D). To determine whether TIMM8A or TIMM13 depletion affects the proliferative capacity of lung cancer cells *in vitro*, CCK-8 assay and colony-formation assay were conducted. As demonstrated in Fig. 4A–C,E,F, silencing of either TIMM8A or TIMM13 significantly reduced cell proliferation. Additionally, cell migration ability *in vitro* was assessed by Transwell assay. The results indicated that knockdown of either TIMM8A or TIMM13 decreased cell migration capacity compared to the control cells (Fig. 4D,G,H). These findings suggest that the TIMM8A-TIMM13 complex may function as an oncogenic factor in lung cancer progression through the regulation of cell proliferation and migration.

### 3.4 Cell Cycle-Related Genes Were Identified as the Downstream Effectors of the TIMM8A-TIMM13 Complex

**Figure 5:**
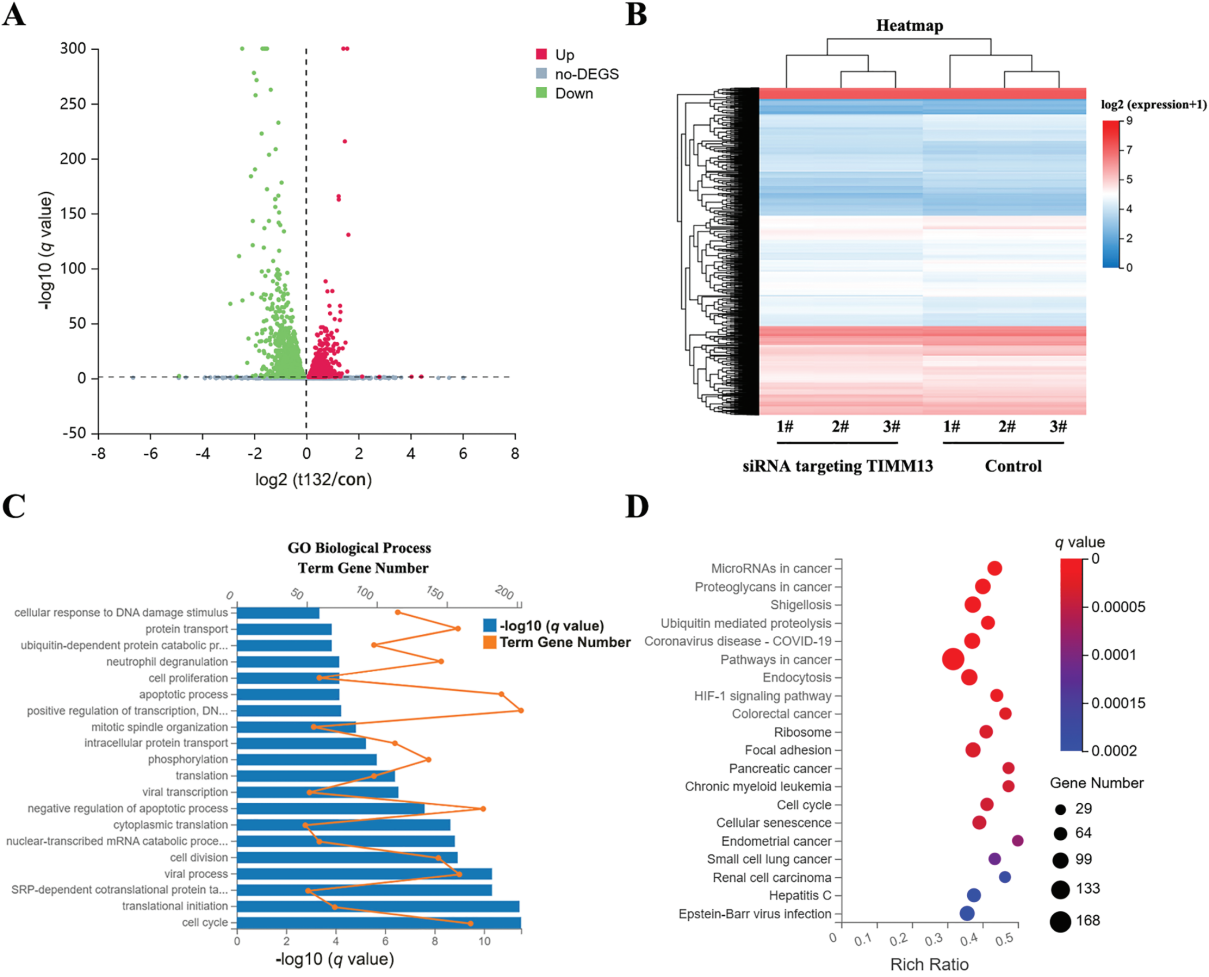
Cell cycle-related genes were identified as the downstream targets of TIMM8A-TIMM13 complex. (**A**) Volcano plot illustrating 1765 upregulated and 2109 downregulated genes between siTIMM13-215-transfected A549 cells and control cells. (**B**) Heatmap depicting mRNA expression profiles comparing the control group and siRNA-TIMM13 transfection group. (**C**) Distribution of differential mRNA expression and -log10 (*q* value) across biological processes following TIMM13 knockdown. (**D**) Kyoto Encyclopedia of Genes and Genomes (KEGG) pathway enrichment analysis of RNA-sequencing data. Enrichment analysis utilized the hypergeometric test (phyper function in R) for *p* value calculation, with false discovery rate (FDR) correction for *q* value determination. Pathways with a *q* value < 0.05 were deemed significantly enriched.

To elucidate the molecular mechanisms by which the TIMM8A-TIMM13 complex regulates lung cancer cell biological behavior, we performed RNA sequencing in siTIMM13-215-transfected A549 cells and their control cells. As shown in Fig. 5A, transfection with siRNA resulted in a differential expression pattern. The volcano plot revealed 1765 upregulated genes and 2109 downregulated genes (Fig. 5A). Cluster analysis demonstrated strong parallelism between the two groups (Fig. 5B). Functional enrichment analysis of genes during biological processes indicated significant alterations in the expression of cell cycle-related molecules after TIMM13 knockdown (Fig. 5C). Further analysis of signaling pathways revealed significant differential regulation of cell cycle-related genes in response to TIMM13 suppression (Fig. 5D).

### 3.5 Disruption of TIMM8A-TIMM13 Complex Resulted in Downregulation of CCND1 and CDK6

**Figure 6:**
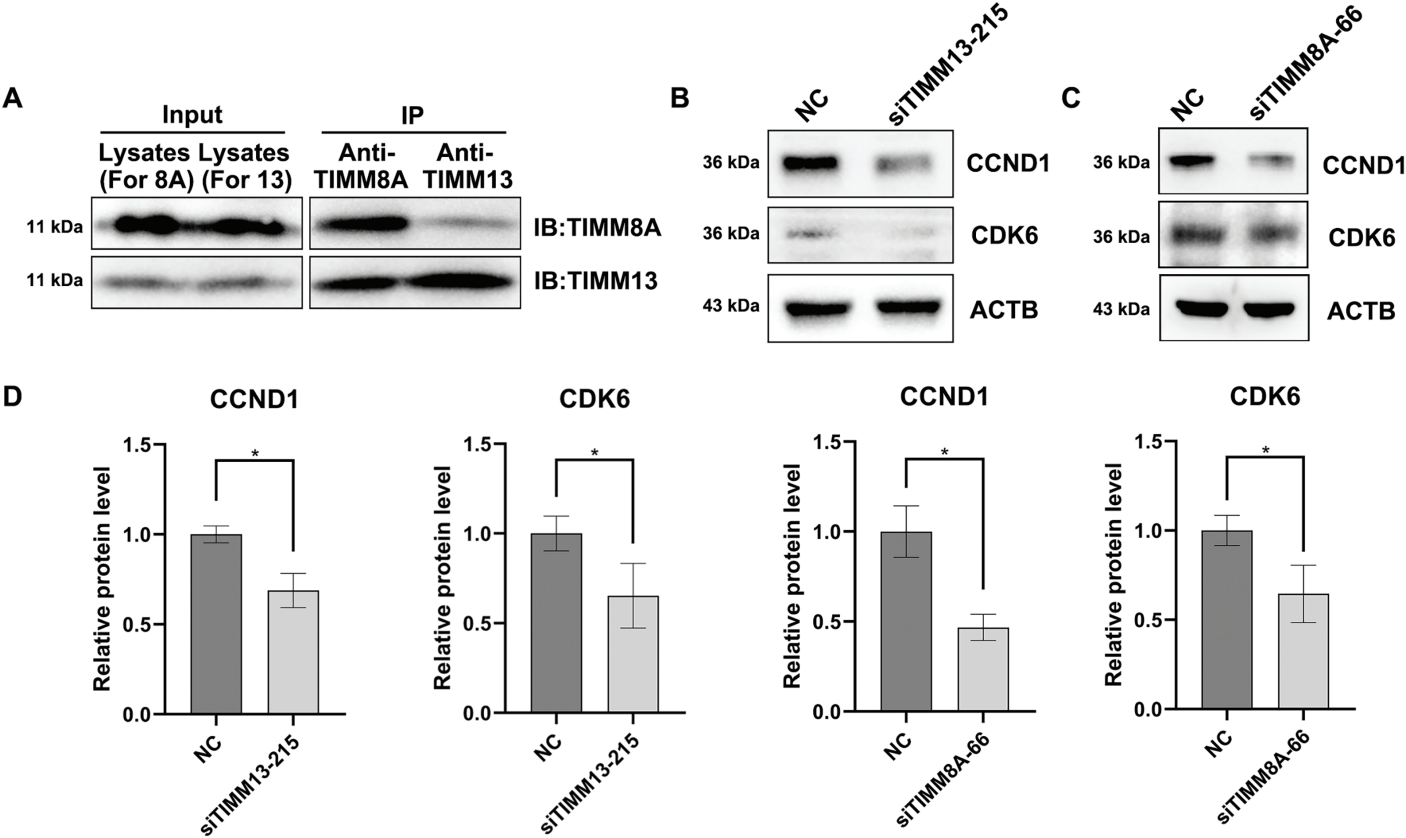
Disruption of TIMM8A-TIMM13 complex resulted in downregulation of CCND1 and CDK6. (**A**) IP assay confirmed that TIMM8A and TIMM13 function as a complex in A549 lung cancer cells. (**B**) CCND1 and CDK6 expression in siTIMM13-215-transfected A549 cells and control cells was verified by immunoblotting. (**C**) CCND1 and CDK6 expression in siTIMM8A-66-transfected A549 cells and control cells was verified by immunoblotting. (**D**) Quantitative analysis of immunoblotting results. Data are presented as mean ± standard deviation (*n* = 3). *p < 0.05 vs. NC

Among these cell cycle-related genes, *CCND1* and *CDK6* showed significant decreases in siTIMM13-215-transfected cells (Table 1). Research has established that CCND1 and its binding partner, CDK6, form an active complex promoting cell cycle progression [24]. Additionally, dysregulation of the CCND1-CDK6 complex frequently leads to aberrant cell proliferation in various human malignancies, including lung cancer [25–27]. Before investigating the biological function of TIMM8A-TIMM13 complex in lung cancer, we confirmed its presence in A549 lung cancer cells through co-IP assay (Fig. 6A). Immunoblotting verified the expression of CCND1 and CDK6 in siTIMM13-215- or siTIMM8A-66-transfected A549 cells compared to control cells. The results demonstrated that inhibition of either TIMM13 or TIMM8A substantially suppressed CCND1 and CDK6 expression (Fig. 6B–D). These findings suggest that disruption of the TIMM8A-TIMM13 complex may suppress lung cancer cell growth through inhibition of the CCND1-CDK6 complex.

**Table 1:** *CDK6* and *CCND1* mRNAs are downregulated in siTIMM13-215-transfected A549 cells compared to their control cells (NC)

	siTIMM13-215 vs. NC	
**Gene**	**Fold change**	**Description**
*CDK6*	0.51683 ± 0.00807	Down
*CCND1*	0.78341 ± 0.02835	Down

## 4 Discussion

Despite ongoing advances in molecular biology and targeted therapy of lung cancer, current therapeutic options, particularly for advanced stages, remain limited. This limitation largely stems from the heterogeneous characteristics of the disease, presenting significant challenges in therapeutic management. Targeted therapy primarily aims to inhibit specific molecules supporting tumorigenesis. The TIMM8A-TIMM13 complex was initially discovered during research on deafness/dystonia syndrome. This complex, primarily located in mitochondria, mediates hTim23 import, and its reduction may significantly contribute to this syndrome [28]. Recent studies have identified overexpression of TIMM8A and TIMM13 in various tumor types, including breast cancer [14–17], endometrial carcinoma [17], osteosarcoma [18], and cutaneous melanoma [19]. Emerging evidence reveals their complicated roles in tumorigenesis and immune modulation. For instance, TIMM8A promotes immune evasion in breast and endometrial cancers by upregulating PD-L1 expression and recruiting immunosuppressive cells such as Tregs and M2 macrophages [16,17]; TIMM13 similarly correlates with CD8^+^ T cell exhaustion and elevated immune checkpoint molecules like PD-1 in melanoma [19], while it primarily exhibits mitochondrial anti-apoptotic functions in osteosarcoma [18]. These context-dependent mechanisms highlight their potential as both therapeutic targets and predictors of immunotherapy response. However, the precise function of this complex in lung cancer cells requires further investigation. The present study elucidates the role of the TIMM8A-TIMM13 complex in lung cancer and highlights its potential as a therapeutic target. Our research demonstrates significant overexpression of both TIMM8A and TIMM13 in lung cancer tissues, correlating with aggressive clinical pathological features and poor prognosis. Functional experiments reveal that complex disruption inhibits lung cancer cell proliferation and migration, indicating its oncogenic role. These findings align with previous studies demonstrating the TIMM8A-TIMM13 complex’s involvement in various malignancies, including breast cancer [14–17], skin cutaneous melanoma [19], and osteosarcoma [18].

Following confirmation of the oncogenic role of the TIMM8A-TIMM13 complex in lung cancer, we investigated its underlying mechanisms. Previous research demonstrated that TIMM13 silencing induced significant mitochondrial dysfunction, leading to ROS production and oxidative injury [18]. However, the precise mechanisms through which the TIMM8A-TIMM13 complex regulated lung cancer progression remained unclear. To address this, RNA sequencing analysis was conducted. The analysis identified significant regulation of ubiquitin, ribosome, focal adhesion, and cell cycle-related pathways following TIMM13 knockdown. Among these, the CCND1/CDK6 complex, a key regulator of lung cancer cell growth, emerged as a potential mediator of TIMM8A-TIMM13 complex oncogenic mechanisms, as TIMM13 or TIMM8A knockdown significantly suppressed CCND1 and CDK6 expression, as confirmed by RNA sequencing data and immunoblotting results. However, co-IP combined Mass Spectrometry experiments revealed no direct interactions between CCND1/CDK6 and the TIMM8A-TIMM13 complex (data not shown), indicating indirect regulatory mechanisms. Considering the crucial role of TIMM8A and TIMM13 in mitochondrial protein import, mitochondrial dysfunction may influence the cell cycle through multiple pathways. For example, compromised mitochondrial function could affect the availability of key metabolite like acetyl-CoA or enhance ROS production, subsequently modulating cell cycle regulators such as CDK6 and CCND1. This indirect regulatory mechanism potentially represents a crucial connection between mitochondrial homeostasis and cell cycle progression [29].

While mitochondrial proteins are traditionally studied for their roles in bioenergetics and apoptosis, emerging evidence suggests they are also involved in the regulation of cell cycle progression [30,31]. Our study reveals that TIMM8A-TIMM13 complex, localized in the mitochondrial intermembrane space, drives lung cancer cell proliferation by upregulation of CCND1 and CDK6, thereby accelerating G1/S transition. This expands the functions of mitochondria in tumorigenesis and raises the possibility of targeting mitochondrial-cell cycle crosstalk for therapy. Although current mitochondrial-targeted therapies primarily focus on disturbing energy metabolism such as OXPHOS inhibitors [32] or inducing cell death via disrupting the inner mitochondrial membrane potential [33], our findings suggest an alternative approach, that is, selectively interfering with mitochondrial proteins like TIMM8A or TIMM13 that regulate cell cycle checkpoints. Future studies may explore whether inhibition of TIMM8A-TIMM13 complex can synergizes with conventional cell cycle inhibitors like CDK4/6 inhibitors in lung cancer treatment.

It must be acknowledged that the present study contains measurable limitations requiring targeted improvements in future work. First, our findings rely primarily on *in vitro* models, and *in vivo* validation using preclinical animal models would strengthen the translational relevance. Second, although clinical correlations were explored, potential confounders, such as age, sex, smoking status or alcohol intake, may influence the interpretation of the prognostic value of TIMM8A-TIMM13 complex. Finally, while we established a link between TIMM8A-TIMM13 complex and CCND1/CDK6 complex, the precise downstream signaling mechanisms remain to be elucidated. Future studies should focus on elucidating the precise molecular mechanisms by which mitochondrial TIMM8A-TIMM13 complex regulates CCND1/CDK6 signaling including transcriptional regulation and post-translational modifications. Additionally, *in vivo* validation using patient-derived xenografts or genetically engineered mouse models would strengthen the clinical relevance of these findings. Moreover, the development of mitochondria-targeted inhibitors against TIMM8A-TIMM13 complex, either alone or in combination with CDK4/CDK6 inhibitors, represents a promising therapeutic avenue worthy of exploration.

## 5 Conclusion

In conclusion, this study demonstrates that components of the TIMM8A-TIMM13 complex exhibit elevated expression and potentially contribute to disease progression and poor outcomes in lung cancer patients through upregulation of CCND1 and CDK6. These findings provide novel insights into the molecular biology of lung cancer development, establishing a foundation for developing TIMM8A-TIMM13 complex-targeted therapies for lung cancer patients.

## Supplementary Materials



## Data Availability

The datasets generated and/or analyzed during the current study are available from the corresponding authors on reasonable request.

## Supplementary Materials

The supplementary material is available online at https://www.techscience.com/doi/10.32604/or.2025.063812/s1.
