# Supplementary material for: TIMM8A-TIMM13 Complex Exerts Oncogenic Functions in Lung Cancer
Source: Oncol Res. 2025 Aug 28;33(9):2435–49. doi: 10.32604/or.2025.063812 (PMC12408856; doi:10.32604/or.2025.063812)
Supplement: Supplementary file 1 [file OncolRes-33-63812-s001.docx]

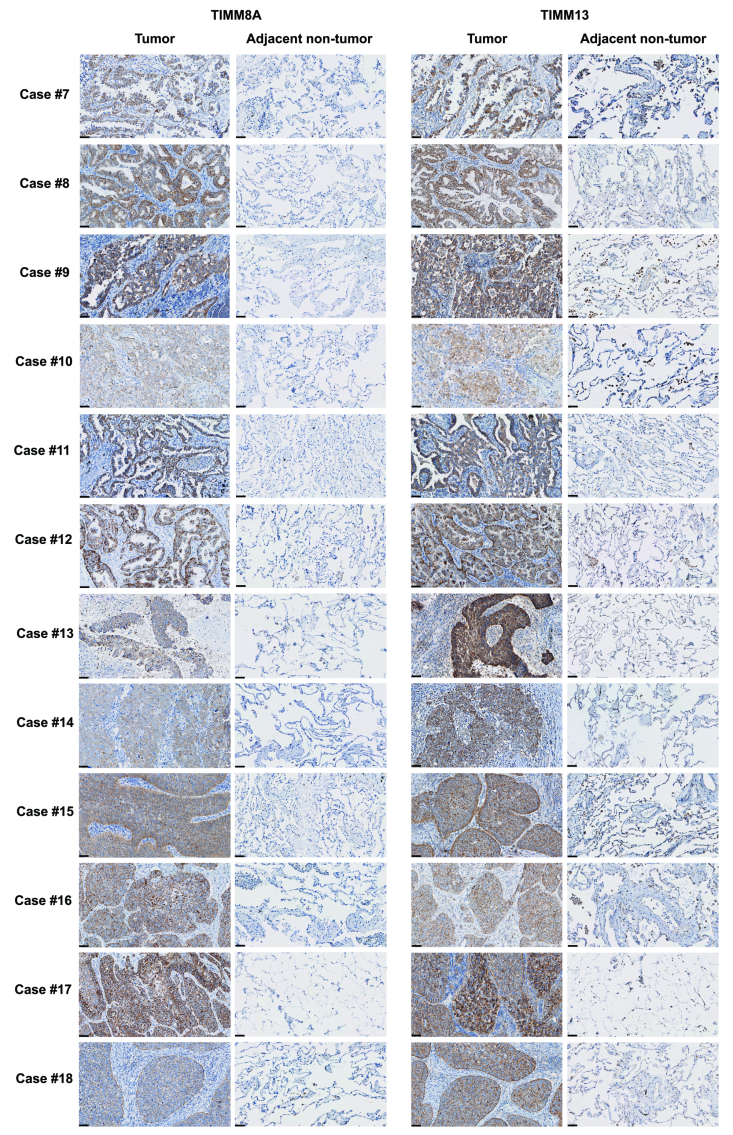


**Supplementary Figure S1:** Verification of TIMM8A and TIMM13 expression in clinical samples. IHC images of TIMM8A and TIMM13 expression in the other 12 lung cancer tissues and paired adjacent normal tissues (Scale bar = 50 μm).
